# Psychological Profiles and Health Status in Japanese Female Patients with Systemic Lupus Erythematosus: The Miyagi Lupus Collaborative Study

**DOI:** 10.2188/jea.12.55

**Published:** 2007-11-30

**Authors:** Yuko Minami, Takeshi Sasaki, Yumiko Arai, Toru Hosokawa, Shigeru Hisamichi, The Miyagi Lupus Study Group

**Affiliations:** 1 Division of Epidemiology, Miyagi Cancer Center Research Institute, 47-1 Nodayama, Medeshima-Shiode, Natori 981-1293, Japan.; 2 Department of Rheumatology and Hematology, Graduate School of Medicine, Tohoku University, 1-1 Seiryomachi, Aoba-ku, Sendai 980-8574, Japan.; 3 Research Unit for Nursing, Caring Sciences and Psychology, National Institute of Longevity Sciences, 36-3 Gengo, Morioka, Obu-shi, Aichi 474-8522, Japan.; 4 Department of Human Development, Faculty of Education, Tohoku University, Kawauchi, Aoba-ku, Sendai 980-8576, Japan.; 5 Department of Public Health, Graduate School of Medicine, Tohoku University, 2-1 Seiryomachi, Aoba-ku, Sendai 980-8575, Japan.; 6 See acknowledgment.

**Keywords:** disease activity, education, health locus of control, personality, systemic lupus erythematosus

## Abstract

Psychological factors have been suspected to be associated with the development of systemic lupus erythematosus (SLE) and patient s health status. However, psychological profiles among Japanese patients with SLE have been poorly understood. We started a prospective study of female patients with SLE in 1995. Using the baseline data from 279 patients in this prospective study, we cross-sectionally analyzed the relations of clinical factors and social factors to psychological factors, and the association between psychological factors and mental and physical health status. We used the Japanese notion *ikigai* as an indicator of mental health, and ambulatory activity as an indicator of their physical health, respectively. To measure psychological factors, the short-form of the Eysenck Personality Questionnaire-Revised (short EPQ-R) and the Multidimensional Health Locus of Control (HLOC) scale were used. Active phase of the disease was significantly related to the neuroticism score in the short EPQ-R. Educational level was inversely related to the scores of powerful others and chance HLOC belief. As for health status, the internal HLOC belief was significantly associated with *ikigai*, and the chance HLOC belief was inversely associated with ambulatory activity. The scores on the short EPQ-R (Extraversion/lntroversion and Neuroticism) were exclusively related to *ikigai*. This study suggests that psychological factors may have effects on both the development of SLE and patient s health status.

## INTRODUCTION

Survival of patients with systemic lupus erythematosus (SLE) has considerably improved during the past three decades^1^^-^^4^^)^. A recent Japanese study shows that the 10-year survival rate among patients with SLE was 93.4%^1^^)^. The concept of SLE might have changed from a rapidly progressive fatal disease to a chronic disease with a favorable prognosis. However, the improvement of survival has been accompanied by the development of impairments and disabilities. The period in which patients survive with disabilities has been prolonged. Consequently, a greater emphasis is being put on psychological profiles and quality of life including health status rather than the improvement of survival in care management for patients with SLE^5^^)^.

Several studies have reported psychosocial profiles among patients with SLE, suggesting that psychological factors might be associated with the onset and exacerbation of disease^6^^-^^14^^)^. Furthermore, recent studies on quality of life show the relation of psychosocial factors to mental and physical health status^7^^,^^15^^-^^20^^)^. It has been recognized that psychological factors may affect not only the development of disease but also patient’s health status. However, most previous findings were from western countries. In Japan, few studies have been conducted^21^^-^^23^^)^. The roles of psychological factors have been poorly understood among Japanese patients.

In order to investigate the associations of psychosocial factors and lifestyle including diet with the development of disease and mortality, we started a prospective study of female patients with SLE in 1995. At baseline, we measured psychological conditions of the patients using two scales: the short-form of the Eysenck Personality Questionnaire-Revised (short EPQ-R) for measuring personality^24^^)^ and the Multidimensional Health Locus of Control (HLOC) scale for assessing belief related to health^25^^)^, and obtained information on a wide range of psychological factors; personality is considered to be a stable psychological factor^26^^)^, whereas belief may be a controllable one^27^^)^. Although the HLOC has been frequently used in studies from western countries^7^^,^^9^^,^^28^^)^, studies based on personality scales are few^14^^,^^29^^)^. The short EPQ-R has never been used in previous studies. Our information may provide not only psychological characteristics in Japanese patients but also additional insights on psychological functioning in SLE. In the present study, we focused on potential effects of these psychological factors mentioned above and analyzed the baseline data. Our hypothesis was that clinical factors such as disease activity and organ damage would be related to psychological conditions measured by the short EPQ-R and the HLOC and that psychological conditions would be associated with health status at the time of data collection among female SLE patients.

## MATERIALS AND METHODS

### Patient selection and procedure

Study subjects were obtained from rheumatology or nephrology departments at 21 hospitals and 2 rheumatology clinics located in northeastern Japan. All these medical institutions are designated referral clinics for SLE. We organized the Miyagi Lupus Study Group with physicians (rheumatologists and nephrologists) in the institutions and started this study.

Female patients with a clinical diagnosis of SLE seen by the physicians during the period from June 1 to September 30, 1995, were recruited. Patients with serious symptoms, e.g. terminal symptoms and severe neuropsychiatric symptoms, were excluded in advance, since a process of data collection might be a burden for them. At the initial visit during this period, the physicians asked female patients without serious symptoms to participate in this study and handed out self-administered questionnaires to them. Of a total of 311 patients whom the physicians asked, 279 patients consented to participation in the study and completed the questionnaires (89.7%). The questionnaire consisted of 9 parts including personal and family histories, psychological and behavioral status, menstrual and reproductive histories and a Food Frequency Questionnaire (FFQ). The FFQ has been validated in a Japanese population^30^^)^.

The patients who gave consent were clinically evaluated. After having performed the physical examination, the physicians completed a form including clinical findings and laboratory data to assess disease activity, organ damage, and medication.

The data obtained from the questionnaire survey and the clinical data from the physicians were combined into baseline data for a prospective study. We extracted several variables for the present study from this baseline data.

### Measures

*Age and social factor* Age at the time of data collection was calculated based on the date of birth. As a social factor, history of education was selected. This is considered to be an appropriate factor for younger SLE patients in the present study. History of education was categorized into two groups (completed or not completed high school, beyond high school).

*Psychological scales* Personality was measured using the short EPQ-R. The short EPQ-R consisting of 48 items is a short version of the EPQ-R, the most recent form of a series of personality inventories developed by Eysenck and colleagues^24^^)^. The scales in the short EPQ-R were labeled Psychoticism (P), Extraversion/Introversion (E), Neuroticism (N) and Lie (L). The Japanese version of the short EPQ-R has been validated by one of the authors (T. H.)^31^^)^; coefficient alpha reliabilities for the four scales (P, E, N and L) in the Japanese version are similar to those in the English version^24^^)^. Test-retest reliability also indicates substantial stability. Although some different interpretations depending on different language and culture could be inevitable, the personality dimensions measured by the short EPQ-R are regarded as applicable to the Japanese population. Coefficient alpha reliabilities among the subjects in the present study were similar to those in the validation study of the Japanese version.

The subjects’ beliefs related to their health were measured by the HLOC scale consisting of 18 items, yielding 3 subscales^25^^)^. Namely, the HLOC evaluates whether individuals regard their health is determined by their own behavior (internality), they regard their health as under the control of powerful others (powerful others), or they believe their health is not controllable by them and in the hands of fate (chance)^25^^,^^27^^)^. Internal consistency in the Japanese version of the HLOC scales has already been confirmed by one of the authors (T. H.) and colleagues (unpublished observation); coefficient alpha reliabilities for the three subscales in the Japanese version are around 0.7. These coefficients were regarded as acceptable. Coefficient alpha reliabilities among the subjects in the present study were also around 0.7 (internality 0.71, powerful others 0.68, chance 0.68).

*Clinical factors* Duration of disease was defined as the period from the date of diagnosis to the date of the data collection. Disease activity was evaluated according to the Lupus Activity Criteria Count (LACC)^32^^)^. Patients with any 2 or more counts were defined as active phase. Organ damage was measured using the Systemic Lupus International Collaborating Clinics/American College of Rheumatology Damage Index (SLICC/ACR DI)^33^^)^, which evaluates cumulative damage due either to the disease, complications of therapy, or intercurrent illness such as cancer. Ocular damage was not counted in the present study, because of the incomplete assessment of ocular lesion. After the exclusion of ocular lesion from the original SLICC/ACR DI, scores on the Damage Index can range from 0 to 44.

*Health status* Since this study was designed to prospectively investigate prognostic factors of SLE, the questionnaire did not include quality of life scales. Therefore, we used the Japanese notion *ikigai* as an indicator of mental health, and ambulatory activity as an indicator of their physical health, respectively. The term *ikigai* is frequently regarded in Japan to denote the sense that life is worth living. *Ikigai* has been used as an indicator of subjective mental health in several studies^34^^,^^35^^)^. The question asked in the questionnaire employed is “Do you have *ikigai?*,” which required a “yes,” “uncertain,” or “no” reply. In subsequent analysis, a “yes” reply was considered to indicate the presence of *ikigai*, and “uncertain” and “no” replies were combined into the absence of *ikigai* category.

Ambulatory activity of each patient was evaluated by a 4-level scale ranging from “completely home-bound” to “free in ambulatory activity.” The patients describing home-bound status (lower two levels) were assigned to the limited ambulatory group, and those describing better ambulation (upper two levels) to the better ambulatory group.

### Statistical analysis

#### Relations of disease activity and organ damage to psychological factors

To investigate the relations of clinical factors (disease activity and organ damage) to psychological factors (personality and health belief), we applied linear regression models^36^^)^. We constructed a model with treating each subscale in the short EPQ-R and the HLOC as a dependent variable. First, considering age and duration of disease as essential control variables in the present study, we estimated the regression coefficients for active phase, greater organ damage, and high educational level with adjustment for only age and duration of disease. Secondly, based on the test statistics for these adjusted regression coefficients, we selected some variables and constructed final regression models for evaluating the relation of clinical factors to psychological factors.

#### Relations of psychological factors to health status

The associations between psychological factors and health status were evaluated using logistic regression models^37^^)^. In the analysis, age, duration of disease, clinical factors, and educational level were selected for control variables, and the odds ratios of the presence of *ikigai* and better ambulatory activity were estimated.

First, we estimated the odds ratios for each subscale of the short EPQ-R and the HLOC with adjustment for only age and duration of disease. Secondly, to estimate the odds ratios adjusted for all control variables, we constructed a model including each subscale of the short EPQ-R and the HLOC, and all control variables. Based on the odds ratios estimated from the models, we evaluated the association of psychological factors with each health status indicator. The analysis of ambulatory activity was conducted only on patients seen at outpatient clinics.

## RESULTS

The distributions of study subjects are shown in Table 1. The duration of disease was unknown in 2 patients. Two hundred and sixty-one patients were from outpatient clinics. Table 2 shows several characteristics of the subjects. Thirty-four patients were diagnosed as active phase. In 4 patients, disease activity was not identified. The mean SLICC/ACR DI score was 1.10±1.07 (SD). Thirty-one patients were classified into the beyond high school group. As for health status, one hundred and fifty-three patients replying yes to the question about *ikigai* were classified into the presence of *ikigai* category. One hundred and twenty-two patients replying uncertain or no (uncertain: 109 patients, no: 13 patients) were summarized into one category: the absence of *ikigai*. Four patients did not reply to the question. Among 261 outpatients, ambulatory activity was limited in 51 patients.

**Table 1.** Distribution of 279 female patients with SLE in the Miyagi Lupus Collaborative Study.

Age (years)	Duration of disease (years)					Total

	0-4	5-9	10-14	15-	Unknown	
-19	9	1	0	0	0	10
20-29	24	34	11	0	0	69
30-39	11	15	10	10	0	46
40-49	20	12	20	23	1	76
50-	12	16	19	30	1	78
Total	76	78	60	63	2	279

**Table 2.** Characteristics among female patients with SLE.

Factor	
Mean age, years	40.6±13.7 (SD)
Mean duration of disease, years	9.9±7.3 (SD)
Active phase, (n)	34
Mean SLICC/ACR DI score	1.10±1.07 (SD)
Beyond high school education, (n)	31

Psychological scales were complete in 250 patients for the short EPQ-R and in 254 patients for the HLOC. Patients completing the scales were slightly younger than those not completing them (data not shown in tables). The subsequent analyses were performed for the patients completing the scales.

### Relations of disease activity and organ damage to psychological factors

First, we estimated regression coefficients for clinical factors and educational level with adjustment for only age and duration of disease. The test statistics for the adjusted regression coefficients indicated that active phase of disease was related to the N score (p=0.0001) and the E score (p=0.0562) in the short EPQ-R and that greater organ damage was related to powerful others HLOC (p=0.0918). Furthermore, the relations of high educational level to powerful others HLOC (p=0.0577) and to chance HLOC (p=0.0490) were also indicated. These imply that disease activity, organ damage, and educational level may explain variations in the scores of the short EPQ-R and the HLOC. Thus, to evaluate relations of active phase, greater organ damage, and other factors to each subscale in the short EPQ-R and the HLOC, we decided to enter all variables selected into a final regression model. The statistics estimated from the final regression model were presented in Table 3 and 4.

**Table 3.** Relations of clinical factors to personality among female patients with SLE.

Factor	P^a)^		E		N		L	
			
	β^b)^	P value	β	P value	β	P value	β	P value
Older age								
	-0.0059	0.4994	-0.0684	0.0001*	-0.0068	0.6669	0.0677	0.0001*
Active phase								
	0.3073	0.3340	-0.9244	0.1372	1.9333	0.0009*	-0.4105	0.3928
Greater organ damage								
	0.0464	0.6485	-0.1357	0.4944	0.2408	0.1908	0.1073	0.4848
Longer duration of disease								
	-0.0238	0.1587	0.0221	0.5026	0.0186	0.5417	0.0023	0.9286
High educational level								
	-0.1271	0.6934	-0.5016	0.4258	0.8266	0.1569	0.2170	0.6559

a) P: Psychoticism; E: Extraversion/Introversion; N: Neuroticism; L: Lie in the short-form of the Eysenck Personality Questionnaire-revised

b) Regression coefficient was adjusted for all other variables.

*: p<0.05

**Table 4.** Relations of clinical factors to multidimensional health locus of control scales among female patients with SLE.

Factor	Internality		Powerful others		Chance	
		
	β^a)^	P value	β	P value	β	P value
Older age						
	0.0135	0.5486	0.0678	0.0010*	-0.0048	0.8428
Active phase						
	-0.9975	0.2352	-0.7668	0.3118	0.1504	0.8677
Greater organ damage						
	-0.0076	0.9769	0.4595	0.0527	0.3052	0.2791
Longer duration of disease						
	-0.0190	0.6517	-0.0491	0.1976	-0.0618	0.1734
High educational level						
	-1.0662	0.2126	-1.5394	0.0467*	-1.8539	0.0445*

a) Regression coefficient was adjusted for all other variables.

*: p<0.05

Table 3 shows the relations of clinical factors, education, and other factors to each scale of the short EPQ-R. Age was positively related to the L score (p=0.0001) and inversely related to the E score (p=0.0001). Active phase of the disease was positively related to the N score (p=0.0009). No relations of organ damage to the short EPQ-R scales were observed.

Table 4 shows the relations of clinical factors, education, and other factors to each scale of HLOC. Age was positively related to the score of powerful others HLOC (p=0.001). Educational level was inversely related to the score of powerful others HLOC (p=0.0467) and chance HLOC (p=0.0445). Organ damage was marginally related to the score of powerful others HLOC (p=0.0527).

### Relations of psychological factors to health status

The associations of psychological factors with health status indicators are shown in Table 5 and 6. The analysis of physical health (i.e., ambulatory activity) was conducted only on outpatients, since inpatients were probably told to stay in bed by doctors. Table 5 shows the odds ratios adjusted for only age and duration of disease. The odds ratios adjusted for age, duration of disease, disease activity, organ damage, and educational level are presented in Table 6. Although the results in both tables are similar, the odds ratios in Table 6 indicate independent associations between psychological factors and health status.

**Table 5.** Odds ratios and 95% confidence interval of psychological factors associated with mental and physical health among female patients with SLE (adjustment for only age and duration of disease).

Factor	*Ikigai ^a)^*		Ambulatory activity ^b)^	
	
	Odds ratio ^c)^	95% confidence interval	Odds ratio ^c)^	95% confidence interval
Eysenck Personality Questionnaire-revised				
Psychoticism (P)	1.12	0.96-1.31	1.02	0.83-1.25
Extraversion/Introversion (E)	1.23	1.12-1.35*	1.06	0.96-1.18
Neuroticism (N)	0.82	0.75-0.90*	0.91	0.81-1.02
Lie (L)	0.99	0.89-1.10	1.01	0.88-1.16

Multidimensional health locus of control				
Internality	1.08	1.02-1.15*	1.00	0.92-1.08
Powerful others	0.94	0.88-1.01	0.96	0.88-1.05
Chance	1.00	0.94-1.05	0.91	0.84-0.99*

a) The term *ikigai* means the sense that life is worth living.

b) Analysis regarding ambulatory activity was performed for the patients seen at outpatient clinics.

c) Odds ratio was adjusted for age and duration of disease.

*: p<0.05

**Table 6.** Odds ratios and 95% confidence interval of psychological factors associated with mental and physical health among female patients with SLE (adjustment for all control variables).

Factor	*Ikigai ^a)^*		Ambulatory activity ^b)^	
	
	Odds ratio ^c)^	95% confidence interval	Odds ratio ^c)^	95% confidence interval
Eysenck Personality Questionnaire-revised				
Psychoticism (P)	1.13	0.96-1.34	1.04	0.84-1.28
Extraversion/Introversion (E)	1.22	1.12-1.34*	1.06	0.96-1.19
Neuroticism (N)	0.83	0.75-0.92*	0.92	0.81-1.03
Lie (L)	0.99	0.89-1.11	1.01	0.88-1.17

Multidimensional health locus of control				
Internality	1.08	1.02-1.16*	1.01	0.93-1.09
Powerful others	0.95	0.89-1.02	0.98	0.89-1.07
Chance	1.00	0.94-1.06	0.92	0.85-1.00*

a) The term *ikigai* means the sense that life is worth living.

b) Analysis regarding ambulatory activity was performed for the patients seen at outpatient clinics.

c) Odds ratio was adjusted for age, duration of disease, disease activity, organ damage, and educational level.

*: p<0.05

Increasing the Extraversion/Introversion score and decreasing that of Neuroticism were significantly associated with *ikigai*. However, none of the scores on the short EPQ-R subscale was related to ambulatory activity. Increasing the score of internal HLOC was associated with *ikigai* and increasing the score of chance HLOC was inversely associated with ambulatory activity.

## DISCUSSION

We cross-sectionally investigated the relations of clinical factors such as disease activity and organ damage to psychological factors, and the associations between psychological factors and health status among about 250 Japanese female SLE patients. Compared the sample size in previous studies ranging from 100 and 200^7^^)^, ours is regarded as a large-scale study. Furthermore, since the patients were derived from referral clinics for SLE, we believe that this study shows psychological characteristics of female SLE patients. However, as several serious patients, possibly inpatients, were dropped from this study, these characteristics may be limited to outpatients. Actually, the analysis restricted to only outpatients showed similar results (data not shown in the tables).

### Relations of disease activity and organ damage to psychological factors

In the present study, several relationships between clinical factors such as disease activity and organ damage, and psychological factors were observed. Furthermore, some differences in the scores of personality and health belief existed among other factors including age and education.

The positive relation of active phase to the N score in the short EPQ-R may be interpreted in two ways: One is to assume that a neurotic personality may be a consequence of the active phase. Another interpretation is that a neurotic personality may exacerbate disease. According to the Eysenck theory, the difference in personality is linked to biological differences in individuals, therefore personality is not easily modulated^26^^,^^38^^)^. In addition, it is known that personality influences the response to stress^39^^,^^40^^)^ and that psychological factors including stress alter the immune system through the brain and endocrine system^13^^,^^41^^-^^43^^)^. If so, it is likely that neurotic personality may serve to exacerbate the disease. Actually, some studies using another psychological scale have suggested that personality or psychological dysfunction might modulate disease outcomes such as disease activity and organ damage^7^^,^^9^^-^^11^^,^^14^^)^. However, there is an opposite result in Japan: Ishikura has reported that disease activity might not be correlated with a psychological feature such as emotional instability among outpatients^23^^)^. There is a possibility that serious diseases may lead to change in personality. The effect of personality on the development of disease should be further investigated using a longitudinal method.

The analysis on the HLOC revealed that patients with lower educational level are likely to regard their health as under the control of powerful others or chance. Since education has been regarded as an important prognostic factor^44^^-^^47^^)^ and HLOC belief is considered to be related to health behavior^25^^)^, our result suggests the possibility that HLOC belief may mediate the relationship between education and prognosis of SLE patients. As for clinical factors, the marginal relation of greater organ damage to increasing powerful others HLOC scores was observed. Previously, Lotstein reported a similar finding^9^^)^. These findings indicate that patients with greater organ damage may require some supports.

### Relations of psychological factors to health status

In the present study, we also investigated another role of psychological factors, i.e., effects on health status. Since we did not use quality of life scales such as the Short Form Health Survey-20 (SF-20)^18^^)^ and the Short Form Health Survey-36 (SF-36)^7^^,^^19^^)^ which have been used in western countries, the present study may not be directly comparable to those in these countries. Nevertheless, *ikigai* is a frequently used term describing subjective mental health in Japan. We consider that the present result may provide a preliminary finding on health status among Japanese patients. Although we cannot find English terms identical with *ikigai*, the concept of this Japanese term may be somewhat comparable to morale, life satisfaction, happiness or self-esteem, from the viewpoint of quality of life^48^^)^. The loss of *ikigai* might lead to learned helplessness, depression, etc.

Increasing the N score in the short EPQ-R was associated with the absence of *ikigai*. In the present study, we have already revealed the relation of disease activity to the N score. Thus, It is likely that neurotic personality may play important roles in both the development of disease and patient’s health status. Besides, the significant association of the E score with *ikigai* was observed. This finding seems to be reciprocal with that in the N score. In western countries, Karlson and Dobkin showed that high stress or psychological distress was associated with worse mental health^7^^,^^20^^)^. It seems that our results may support these findings.

Some HLOC subscales were significantly associated with health status. The association between increasing internal HLOC score and *ikigai* may indicate that the belief that patients’ health is determined by their own behavior may make them feel life is worth living. HLOC belief has been considered to play a significant role in changing health behavior^25^^)^. Therefore, if patients were to adjust their internal HLOC beliefs adequately, they could improve their mental health by practicing healthy behaviors. On the other hand, the association between increasing chance HLOC score and limited ambulatory activity may imply that patients staying at home are likely to believe that their health is determined by chance. It is possible that health status may influence HLOC beliefs.

As mentioned above, we have explained patient’s health status based on subjective scales. Recently, health status scales such as SF-36^49^^)^ and the Arthritis Impact Measurement Scales, version 2 (AIMS2)^50^^)^ have been validated in a Japanese population and may be available in Japan. A quality of life scale for Japanese patients with intractable disease including SLE was also developed^51^^)^. It is necessary to confirm our findings, using these validated scales, in future studies.

In the present study, we discussed the results based on two hypotheses; age, educational level and clinical factors may be related to psychological factors such as personality and health belief, and psychological factors in turn may be associated with health status of Japanese female patients with SLE. Although additional analysis showed some weak correlations among subscales in the short EPQ-R and the HLOC (Table 7), we did not take these correlations into consideration. Recently, McKinley has proposed an application of a structural equation model for explaining contributors to health problems in SLE patients^52^^)^. This proposed method may enable us to explain correlations among clinical factors, personality, health belief, and health status based on a single model. However, in order to clarify psychological profiles in Japanese SLE patients, we believe that we should investigate independent associations of psychological factors with the development of disease and health status first. Applications of the new statistical model will be attempted in future studies.

**Table 7.** Pearson correlation coefficients among subscales in the EPQ-R ^a)^ and the HLOC ^b)^.

EPQ-R	Correlation coefficient		

	HLOC		

	Internality	Powerful others	Chance
Psychoticism (P)	0.0255	-0.1359	0.1015
	(0.6959)	(0.0365)*	(0.1191)
Extraversion/Introversion (E)	0.1591	0.0315	-0.0035
	(0.0142)*	(0.6300)	(0.9570)
Neuroticism (N)	-0.0461	0.0745	0.0518
	(0.4800)	(0.2534)	(0.4272)
Lie (L)	-0.0448	0.0520	-0.0542
	(0.4924)	(0.4256)	(0.4059)

a) EPQ-R: Eysenck Personality Questionnaire-revised

b) HLOC: Multidimensional health locus of control

( ): p-value for a correlation coefficient

*: p<0.05

There are some limitations in this study which require comment. First, although lifestyle or socioeconomic factors and medication may also affect psychological conditions and health status, we did not include most of these factors in the present analysis. Since there is a possibility that clinical conditions (disease activity, organ damage), i.e., main study variables may alter patient’s lifestyle and medication, it seems difficult to identify appropriate lifestyle factors. In the present study, we selected only educational level that is a stable socioeconomic factor. Relations among lifestyle factors, medication, clinical factors, and health status are supposed to be more complicated.

Secondly, since present findings were based on a cross-sectional analysis, causality cannot be certain. Although our previous case-control study^53^^)^ showed the effects of dietary and reproductive factors on the development of SLE, the data on psychological conditions were not included in that study. Now we are conducting a follow-up of the 279 female patients. The temporal relation of psychological factors to the development of disease may be clarified by this longitudinal study.

In summary, our findings suggest that psychological factors may play roles in not only the development of disease but also health status in female SLE patients. We believe that this study is the first step for clarifying psychological profiles and health status in Japanese patients. To disentangle complicated relations among psychological factors, health status, clinical factors, and other factors, more research is needed.
